# Perineuronal net degradation in aggressive glioblastomas with *KANK1::NTRK2* fusions

**DOI:** 10.1093/noajnl/vdag219

**Published:** 2026-08-20

**Authors:** Madhumala K Sadanandappa, Nishika Karbhari, Brizha Knowles, Scott M Palisoul, Edward G Hughes, Laura J Tafe, George J Zanazzi, Chun-Chieh Lin, Jennifer Hong

**Affiliations:** Department of Pathology and Laboratory Medicine, Dartmouth-Hitchcock Medical Center, Lebanon, New Hampshire, USA; Department of Neurology, Dartmouth-Hitchcock Medical Center, Lebanon, New Hampshire, USA; Department of Neurology and Medical Oncology, Mayo Clinic, Rochester, Minnesota, USA (N.K.); Geisel School of Medicine at Dartmouth, Hanover, New Hampshire, USA (B.K.); Department of Pathology and Laboratory Medicine, Dartmouth-Hitchcock Medical Center, Lebanon, New Hampshire, USA; Department of Pathology and Laboratory Medicine, Dartmouth-Hitchcock Medical Center, Lebanon, New Hampshire, USA; Department of Pathology and Laboratory Medicine, Dartmouth-Hitchcock Medical Center, Lebanon, New Hampshire, USA; Department of Pathology and Laboratory Medicine, Dartmouth-Hitchcock Medical Center, Lebanon, New Hampshire, USA; Dartmouth Cancer Center, Dartmouth-Hitchcock Medical Center, Lebanon, New Hampshire, USA; Department of Pathology and Laboratory Medicine, Dartmouth-Hitchcock Medical Center, Lebanon, New Hampshire, USA; Dartmouth Cancer Center, Dartmouth-Hitchcock Medical Center, Lebanon, New Hampshire, USA; Dartmouth Cancer Center, Dartmouth-Hitchcock Medical Center, Lebanon, New Hampshire, USA; Department of Surgery, Section of Neurosurgery, Dartmouth-Hitchcock Medical Center, Lebanon, New Hampshire, USA

**Keywords:** extracellular matrix, gliomas, *NTRK* gene fusion, spatial multi-omics, tumor microenvironment

## Abstract

**Background:**

Approximately 10% of glioblastomas harbor targetable genomic fusions. *NTRK2* participates in a variety of fusion events that drive tumorigenesis. Two previous reports have described *KANK1::NTRK2* fusions in adult glioblastoma patients with poor survival.

**Methods:**

We performed a retrospective analysis of glioblastoma patients treated at Dartmouth-Hitchcock Medical Center (DHMC) from 2020 to 2025 to identify cases harboring *KANK1::NTRK2* fusions. Clinical presentation, treatment, histopathologic features, and outcomes were reviewed. In addition, we conducted GeoMx whole-transcriptome and high-plex proteomic digital spatial profiling of a *KANK1::NTRK2*-positive glioblastoma and a comparator tumor from a long-term survivor. Candidate biomarkers were orthogonally validated using immunohistochemistry and/or immunofluorescence.

**Results:**

Two patients with *KANK1::NTRK2* fusion glioblastoma were identified, both demonstrating rapid progression, therapeutic resistance, and survival of less than 7 months. Proteomic profiling showed increased expression and activation of canonical NTRK2 downstream signaling pathways, particularly MEK1/2 and ERK1/2. This was accompanied by upregulation of extracellular matrix remodeling enzymes, including MMP3, MMP14, and ADAM15, along with reduced expression of extracellular matrix-associated transcripts and perineuronal net components in particular compared to a non-fusion glioblastoma.

**Conclusions:**

These limited, hypothesis-generating findings suggest constitutive NTRK2 signaling may promote coordinated extracellular matrix degradation and remodeling, potentially facilitating rapid and aggressive tumor growth and invasion in a subset of glioblastomas.

Key Points

- Rare *KANK1::NTRK2* fusion is associated with high-grade gliomas and may function as an oncogenic driver.
- Co-occurring genomic alterations and upregulation of oncogenic signaling pathways likely contribute to tumor aggressiveness.
- Enhanced degradation of the brain extracellular matrix may underlie tumor infiltration, rapid progression, early recurrence, and poor survival.

Importance of the Study

Glioblastoma is the most common malignant intracranial tumor and is associated with a median survival of approximately 15 months; however, a subset of patients exhibits rapidly progressive disease with therapeutic resistance and substantially shorter survival. The molecular and cellular mechanisms driving these highly aggressive tumors remain poorly understood. Here, we report 2 cases of glioblastoma harboring rare *KANK1::NTRK2* fusions, both with survival of less than 7 months. Comparative spatial whole-transcriptomic and high-plex immune-oncology proteomic (577 proteins) analyses, using a non-fusion glioblastoma from a long-term survivor as a reference, demonstrated increased MAP kinase pathway activity and marked disruption of extracellular matrix organization. Remarkably, perineuronal nets were degraded in glioma-adjacent regions. These findings underscore the importance of deep molecular phenotyping in short-term survivors and identify extracellular matrix alterations as potential biomarkers of aggressive glioblastoma biology.

Glioblastoma (GBM) is the most common malignant primary brain tumor in adults and is characterized by rapid progression, diffuse infiltration, and resistance to therapy. Despite standard-of-care treatment, median overall survival remains approximately 12-15 months, although a subset of long-term survivors (>5 years) is well documented.^1^ At the other end of the clinical spectrum, however, are highly aggressive GBMs associated with extremely rapid progression and early mortality, which remain underrepresented in the literature. Accordingly, there is a paucity of data explaining the aggressiveness of these particularly lethal tumors in short-term survivors, defined by survival of less than 6 months from diagnosis.^2^

Over the past 2 decades, advances in molecular characterization of gliomas have significantly influenced prognosis and clinical management. Specific genetic alterations, including *IDH* mutation^3^ and *MGMT* promoter methylation,^4^ have been strongly associated with improved survival outcomes. In contrast, the molecular features underlying exceptionally aggressive GBMs—and the mechanisms driving their growth and invasiveness—remain poorly understood.

Here, we report 2 cases of highly aggressive GBMs harboring *KANK1::NTRK2* fusions in patients who survived less than 7 months despite standard-of-care treatment, including craniotomy with gross total resection (Table 1). Fusions involving *NTRK* genes have been identified across a range of central nervous system (CNS) tumors. The 3 *NTRK* genes encode transmembrane receptor tyrosine kinases that play critical roles in neural development. Oncogenic *NTRK* fusions promote tumorigenesis through constitutive activation or overexpression of tyrosine kinase signaling pathways, leading to enhanced cellular proliferation and resistance to apoptosis.^12^ Notably, different *NTRK* family members are associated with distinct prognostic implications: *NTRK1* fusions have been linked to more favorable outcomes, whereas *NTRK2* fusions have been associated with poorer prognosis.^13^

**Table 1.** Published cases of *KANK1::NTRK2* fusions in gliomas

Reference	Age (yr)/gender	Location	Diagnosis	Treatment	Outcome
Lopez et al (2019)^5^	2/F	Right cerebellum	Pilocytic astrocytoma	Gross total resection	Recurrence and anaplastic progression at 10 mo
Magnum et al (2021)^6^	6/M	Right frontal horn with leptomeningeal disease	Anaplastic ependymoma	Resection, proton craniospinal irradiation to a dose of 39.6 Gy followed by primary tumor bed boost to a total dose of 54 Gy	Recurrence at 7 mo, Larotrectinib treatment with clinical and radiographic response at 17 mo.
Shepherd et al (2021)^7^	26/M	Left frontal lobe	Glioblastoma	Subtotal resection, 6-week temozolomide and concurrent radiation therapy (200 cGy per fraction, 30 total fractions), followed by treatment with Larotrectinib	Recurrence at 106 days
Ali et al (2024)^8^	NA/NA	Left temporal lobe	Infant-type hemispheric glioma	Combination chemotherapy	Recurrence at 21 mo
Kim et al (2024)^9^	72/M	Right temporal lobe	Glioblastoma	Resection	Death at 4 mo
Oqbani et al (2025)^10^	6/F	Left parietal lobe with extension into lateral ventricle	Glioneuronal tumor with ATRX alteration, kinase fusion, and anaplastic features	Subtotal resection, whole brain radiotherapy 30 Gy/10 fractions with concurrent oral etoposide	Variable response at 5 mo
Akrout et al (2025)^11^	19/F	Right frontal lobe with contralateral extension	Glioneuronal tumor with ATRX alteration, kinase fusion, and anaplastic features	Subtotal resection, cisplatin and etoposide, whole brain irradiation (30.6 Gy) with 23.4 Gy boost to tumor bed	Progression at 26 mo. Entrectinib treatment with complete radiographic response at 40 mo

Abbreviations: F = female; M = male; NA = not available; Gy = gray; cGy = centigray; mo = month; yr = year.

CNS neoplasms harboring *KANK1::NTRK2* fusions are exceedingly rare, with only 7 previously reported cases, all of which progressed to high-grade gliomas.^5-11^ To further understand the molecular pathogenesis of these aggressive tumors, we performed spatial whole-transcriptome and high-plex proteome profiling of glioblastoma with *KANK1::NTRK2* fusion. The resulting data were analyzed using differential expression and pathway enrichment, with comparisons to a glioblastoma lacking this fusion. Key findings were subsequently validated by immunohistochemistry and/or immunofluorescence. Additionally, to investigate the potential mechanism contributing to the aggressive clinical behavior and marked local invasiveness of these tumors, we assessed degradation of extracellular matrix (ECM) components in the tumor microenvironment, including perineuronal nets (PNNs), which are specialized ECM surrounding inhibitory interneurons that play key roles in the regulation of synapse development, protection of neurons from oxidative stress,^14^ and have been implicated in glioma invasion.^15^

## Methods

### Patients and Tissues

Two glioblastoma cases harboring *KANK1::NTRK2* fusions were included in this study: a 62-year-old man diagnosed in 2023 (case #1) and a 76-year-old woman diagnosed in 2020 (case #2). A comparative non-fusion GBM case in a 73-year-old man diagnosed in 2022 with 29 months of survival, harboring a pathogenic *NF1* p. S47Ffs*20 alteration and unmethylated *MGMT* promoter (data not shown) was also included. Formalin-fixed, paraffin-embedded (FFPE) tissue blocks were used for all histologic and molecular analyses, which were performed in a College of American Pathologists (CAP)-accredited and Clinical Laboratory Improvement Amendments (CLIA)-certified laboratory at the Dartmouth-Hitchcock Medical Center (DHMC).

### Histologic Analysis

FFPE tissue sections (4 or 10 µm) were stained with hematoxylin and eosin (H&E) or subjected to immunohistochemistry (IHC) using antibodies against GFAP (clone GA5, ready-to-use, Leica Biosystems, Deer Park, IL), Ki-67 (clone MIB-1, 1:70, Agilent Technologies, Carpinteria, CA), pan-NTRK (clone EPR17341, 1:500, Abcam, Waltham, MA), S100B (code number GA504, ready-to-use, Agilent Technologies, Carpinteria, CA), phospho-MEK1 (clone ab214445, 1:100, Abcam), and phospho-ERK1/2 (clone SP327, 1:100, Abcam). Slides were scanned using an Aperio image scanner (Leica Biosystems, Deer Park, IL), and images were acquired using Aperio eSlide Manager.

### Radiological Analysis

Patients underwent standard volumetric 1.5-3 T magnetic resonance imaging (MRI) with and without gadolinium at initial diagnosis and at regular intervals following surgical resection and adjuvant therapy for clinical surveillance.

### Molecular Analysis

DNA and RNA were extracted from FFPE tissue using the Qiagen AllPrep DNA/RNA FFPE Kit (Qiagen, Germantown, MD) according to the manufacturer’s protocol. Chromosomal copy number variations (CNV), monosomy, and loss of heterozygosity were assessed using the OncoScan CNV Assay (Thermo Fisher Scientific, Waltham, MA),^16^ a single nucleotide polymorphism-based chromosomal microarray platform. Targeted RNA sequencing for gene fusions was performed using the TruSight Tumor 170 assay on the NextSeq 500 system (Illumina, San Diego, CA).^17^

### Spatial Profiling

Spatial proteogenomic profiling of case #1 was performed using the GeoMx digital spatial profiler (DSP) (Bruker Spatial Biology, Bothell, WA) with sequencing readout on an Illumina platform. FFPE tissue from the initial temporal lobe resection was used to construct a tissue microarray (TMA) with 0.6 mm cores and sectioned at 5 μm. Slides were processed according to the manufacturer’s protocol (Bruker Spatial Biology) using a Leica BOND Rx autostainer and probed with 2 high-plex panels: the Whole Transcriptome Atlas (WTA; >18,000 transcripts, #121401102) and the Immuno-Oncology Proteome Atlas (IPA; > 570 proteins, #121300160).^18^

For tissue visualization and region of interest (ROI) selection, slides were co-stained with SYTO13 (nuclear stain; #531-121300303, Bruker Spatial Biology) and pan-cytokeratin (PanCK; #531-121300320) (from the GeoMx Solid Tumor TME Morphology Kit, #531-121300310, Bruker Spatial Biology) and aggrecan (ACAN; 1:100, clone EPR14664, #ab232628, Abcam, and goat anti-rabbit IgG Alexa Fluor 647; 1:400, #A21245, Thermo Fisher Scientific). Based on H&E-guided histology review, 250 μm ROIs were selected by a board-certified neuropathologist (G.Z.). UV-cleavable barcodes conjugated to probes (WTA panel) and antibodies (IPA panel) were released from selected ROIs, PCR-amplified, pooled, and sequenced (27 bp paired-end reads) on the Illumina NovaSeq 6000 using S1 Reagent Kit v1.5 (100 cycles).

FASTQ files were processed using the GeoMx-NGS Pipeline (v3.1.1.6) to generate digital count conversion (DCC) files. Data were analyzed using the GeoMx DSP Data Analysis Suite with default quality control thresholds, including raw reads (>1000), aligned reads (>80%), sequencing saturation (>50%), no-template control (<1000), and nuclei counts per ROI. RNA data were normalized using Q3 normalization, whereas protein data were normalized using housekeeping targets (GAPDH, Histone H3, TOMM20, RPS6, and Calreticulin). Differential expression was performed using linear mixed-effects models with Benjamini-Hochberg correction for multiple testing of fusion (*n* = 8 ROIs) and non-fusion (*n* = 5 ROIs) with adjusted random effects for scan ID, sample ID, and cores.

### Immunostaining

FFPE blocks from both cases were retrieved from the neuropathology archive, sectioned at 5 μm on a rotary microtome, and mounted onto SuperFrost Plus charged glass slides (Thermo Fisher Scientific). Sections were deparaffinized and rehydrated through a standard graded series at room temperature: xylene (3 × 5 min), 100% ethanol (2 × 5 min), 95% ethanol (2 × 5 min), 70% ethanol (2 × 5 min), and deionized water (2 × 5 min).

A hydrophobic barrier was applied using a PAP pen. Sections were blocked in phosphate buffer solution (PBS) containing 10% normal donkey serum (code: 017-000-001, Jackson ImmunoResearch, West Grove, PA) and 0.4% Triton X-100 (Sigma-Aldrich) for 30 min at room temperature in a humidified chamber. Perineuronal nets (PNNs) were labeled by incubation with unconjugated *Wisteria floribunda* agglutinin (WFA; 1:1000 in blocking buffer, #L-1350-5, Vector Laboratories, Newark, CA) overnight at 4°C in a humidified chamber.

Sections were washed with PBST (3 × 5 min, room temperature) and incubated with Cy3-conjugated streptavidin (1:200 in blocking buffer, catalog #SA-1300-1, Vector Laboratories, Newark, CA) overnight at 4°C in a humidified dark chamber. Nuclear counterstaining was performed with 4ʹ,6-diamidino-2-phenylindole (DAPI; 0.1 μg/mL in PBS, #D9542, Sigma-Aldrich, St. Louis, MO) for 10 min at room temperature, followed by two 5 min PBS washes.

Slides were briefly air-dried, cover-slipped with ProLong Gold Antifade Mountant (Thermo Fisher Scientific), and cured overnight at room temperature in the dark before storage at 4°C. Fluorescence imaging was performed on a Nikon Eclipse Ti-3 spinning disk confocal microscope using a 20x objective. Acquisition parameters such as laser power, exposure time, and gain were held constant within each experimental run. Z-stack images were acquired and exported as .nd2 files for downstream analysis. 20× confocal microscopy images of tumor sections from GBM case #1 and GBM case #2 were tiled to generate complete sections for each case. Representative images from regions at the tumor brain interface were identified and selected for inclusion in a non-blinded manner.

### Secondary Analysis of Public Data

Ivy Glioblastoma Atlas Project (IvyGAP) data were queried to identify potential control tumors for comparator analysis.^19^ Nine Neoplasms with unmethylated *MGMT* promoters and no EGFR amplification were identified (Supplementary Figure S5A). Of these, only 3 tumors with leading-edge transcriptomes were included, as this was the region of interest in our spatial transcriptomic data for GBM case #1. For comparative analysis, gene expression values, calculated using log2 normalization, were analyzed for IvyGAP-leading edge, infiltrative tumor, and cellular tumor and *KANK1::NTRK2* GBM case #1.

## Results

### Clinico-Pathologic Characterization of *KANK1::NTRK2* GBMs

We report 2 adult patients with glioblastomas harboring *KANK1::NTRK2* fusions, both exhibiting aggressive, rapidly progressive clinical courses.

#### Case #1

**Figure 1.**
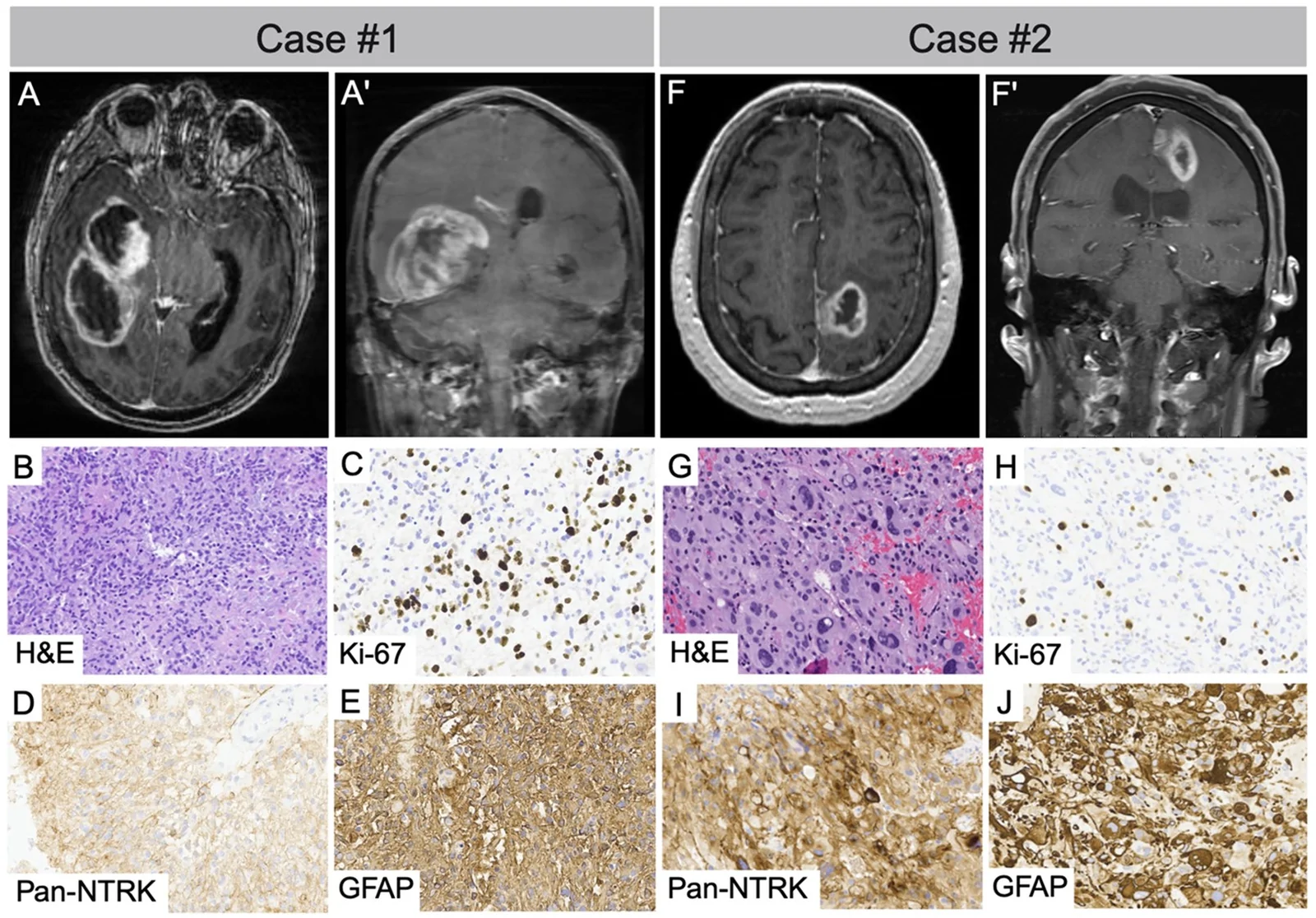
Radiologic and histologic characterization of *KANK1::NTRK2* fusion GBMs. Case #1 (A-E) and case #2 (F-J): preoperative T1-weighted MRI with contrast (A, F) axial and (A′, F′) coronal views. (A, A′) Case #1 shows a 9.4 × 5 × 4.8 cm right temporal mass with significant mass effect, including a 1.2 cm midline shift, uncal herniation, and brainstem compression, whereas (F, F′) case #2 exhibits a 3 × 4 × 2.9 cm left frontoparietal junction mass that is rim-enhancing with central necrotic area. (B, G) Hematoxylin and eosin-stained (H&E) sections reveal highly pleomorphic tumor cells with irregular, hyperchromatic nuclei. (C, H) Both tumors show abundant Ki-67 antibody labeling, indicating high proliferative activity. (D, I) Pan-NTRK immunoreactivity and (E, J) positive GFAP staining were observed in both cases.

**Figure 2.**
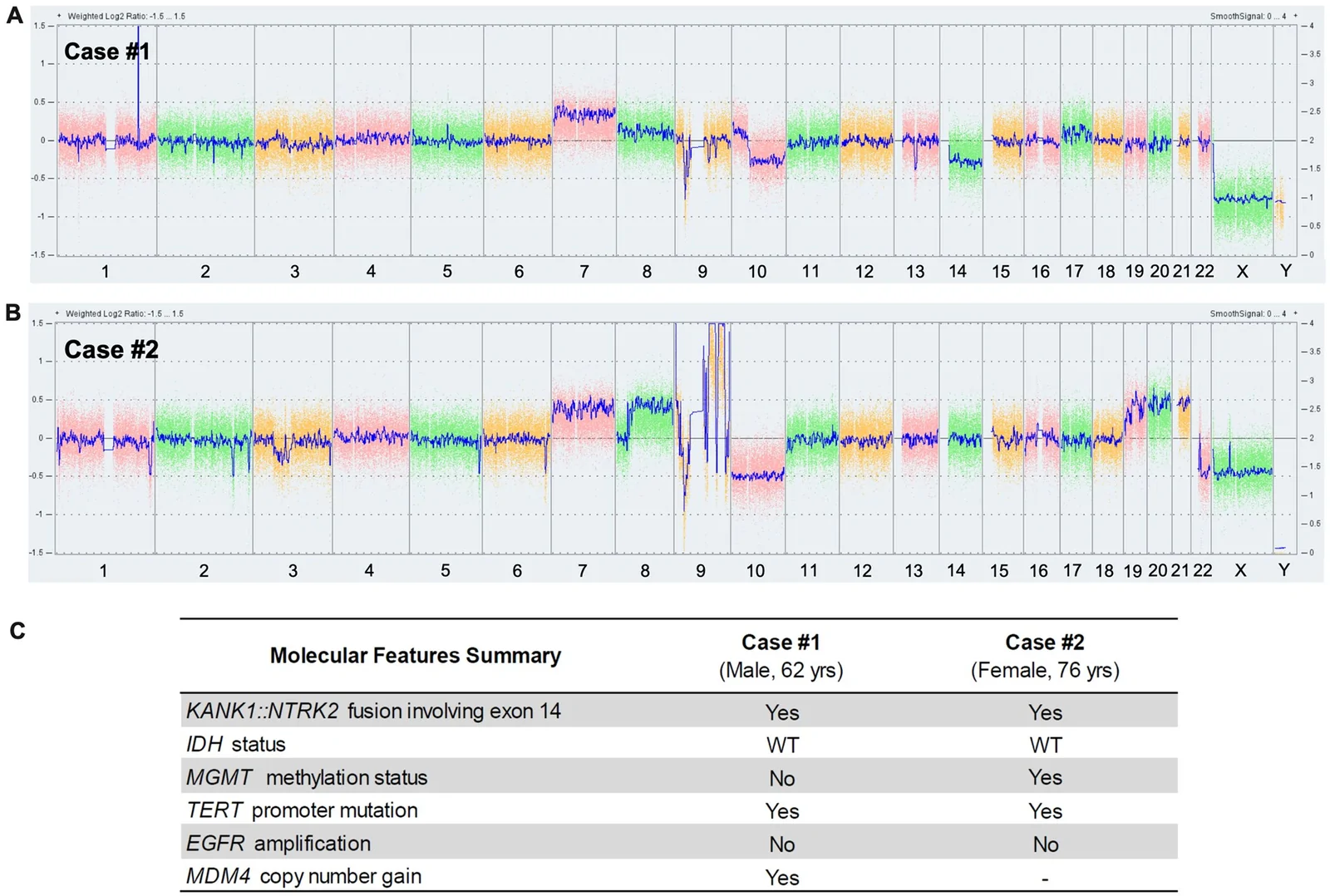
Molecular characterization of *KANK1::NTRK2* fusion GBMs. Copy number variation profiles for (A) case #1 and (B) case #2 show multiple genomic alterations, including chromosome 7 gains and chromosome 10 losses. (C) Summary of genomic alterations for the 2 cases using the TruSight Tumor 170 next-generation sequencing assay.

**Figure 3.**
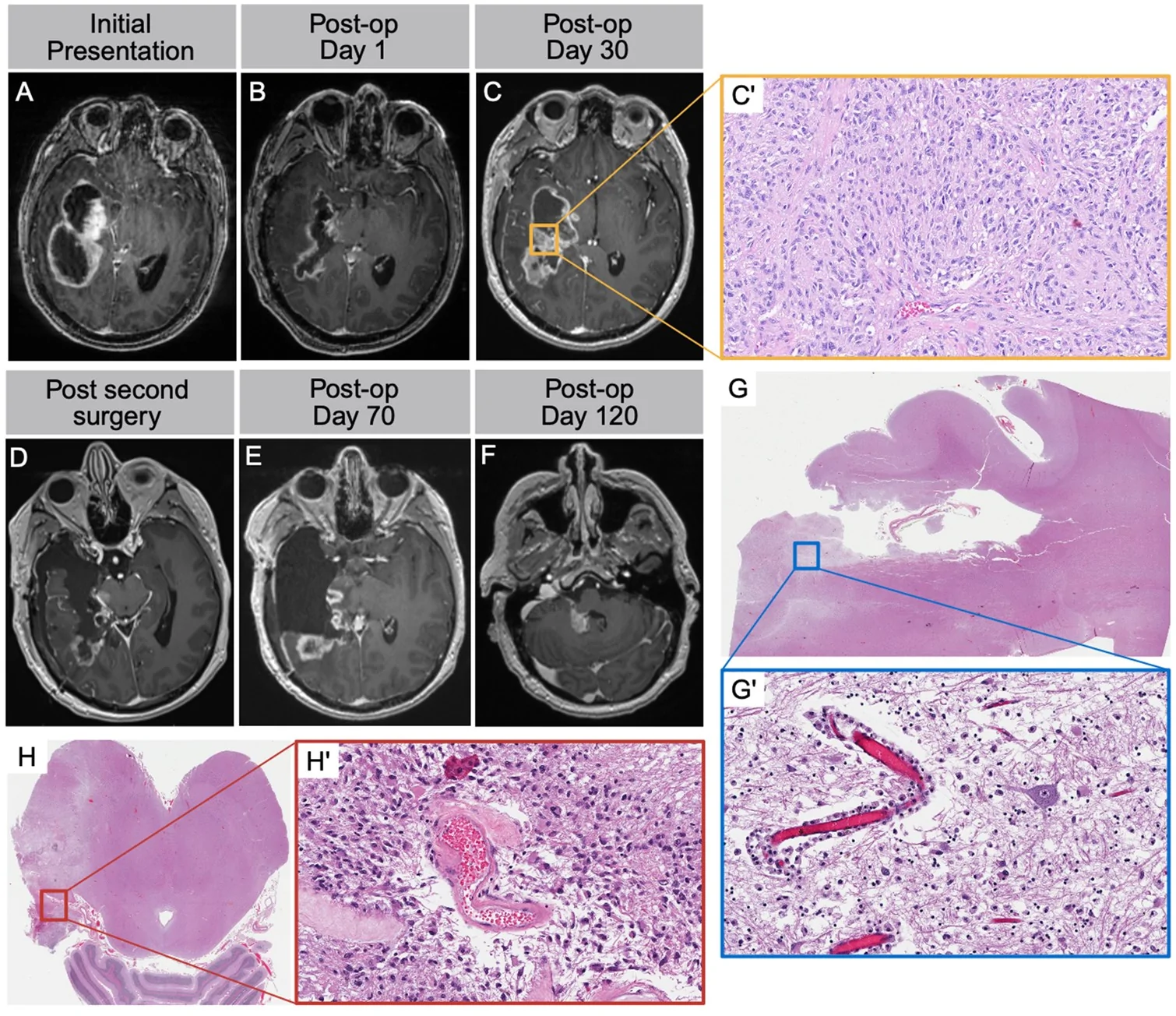
Clinical and radiographic course of case #1. (A-F) Axial T1-weighted postcontrast MRI images demonstrating disease evolution. (A) Pre-operative MRI (reproduced from Figure 1A) showing a large, centrally necrotic mass in the deep right temporal lobe. (B) Postoperative imaging obtained 1 day after initial debulking surgery. (C) Imaging at 30 days postoperatively shows increased peripheral enhancement at the margin of the resection cavity, consistent with early tumor progression. (D) MRI obtained 1 day after repeat resection demonstrated residual enhancement in the right cerebral peduncle and along the posterior margin of the right resection cavity. (E) Follow-up imaging demonstrates recurrent peripheral enhancement at the resection cavity, consistent with continued tumor progression on post-operative day 70. (F) A new enhancing lesion is present in the right cerebellar hemisphere on post-operative day 120, shortly before the patient was transferred to hospice. (G, G′) Low- and high-magnification H&E-stained sections of the right temporal lobe resection cavity at autopsy, showing prominent treatment effects. (H, H′) Low- and high-magnification H&E-stained sections of the midbrain at autopsy, demonstrating infiltrative glioblastoma.

A 62-year-old man who initially presented to the DHMC Parkinson’s Disease and Movement Disorder Center with abnormal tongue movements described as a clicking against the roof of the mouth, accompanied by new mid-frontal and occipital headaches over the preceding 3 months. The tongue movements were subsequently interpreted as focal seizures, and treatment with Keppra (Levetiracetam) was initiated. Neurologic examination revealed left-sided hyperreflexia, bradykinesia, and increased tone, and Karnofsky Performance Status (KPS) was rated as 70. Brain MRI, obtained to evaluate the new headaches and rule out lesions of the Guillain-Mollaret triangle, revealed an enhancing right temporal mass measuring 9.4 × 5 × 4.8 cm, with significant mass effect, including a 1.2 cm midline shift, uncal herniation, and brainstem compression (Figures 1A-A′ and 2A). The patient received mannitol and dexamethasone and underwent 5-aminolevulinic acid (5-ALA)-guided subtotal resection of the tumor the following day (Figure 3B).

Histopathologic examination demonstrated a gemistocytic astrocytoma with palisading necrosis (Figure 1B). The tumor exhibited a high Ki67 proliferative index (approximately 40%), immunoreactivity for pan-NTRK, and diffuse GFAP positivity (Figure 1C-E). Molecular profiling revealed an unmethylated *MGMT* promoter, *TERT* promoter mutation (c.-124C > T; VAF of 25%), *PTEN* variant (c.254-1del; VAF of 28.89%), *MDM4* amplification (6 copies), no EGFR amplification, and a *KANK1::NTRK2* fusion involving exon 14, preserving the full NTRK2 kinase domain (amino acids 555-822) (Figure 2A).

The patient-initiated radiation therapy (60 Gy in 30 fractions) with concurrent temozolomide, followed by planned adjuvant temozolomide. Despite these interventions, he presented 1 month later with worsening headaches and emesis. MRI demonstrated local tumor progression with increased mass effect (Figure 3C). A second debulking procedure confirmed a recurrent glioblastoma (Figure 3C′). Targeted therapy with the NTRK inhibitor entrectinib (400 mg daily) was subsequently initiated. Tumor progression was observed 10 weeks later, and a right cerebellar lesion was removed by laser interstitial thermal therapy (Figure 3E and F). The patient was admitted to hospice 1 month afterward and passed 7 months after initial diagnosis. Autopsy brain donation was performed for research purposes (Figure 3G and H).

#### Case #2

A 76-year-old right-handed woman presented with a right foot drop, and 1 month of progressive right lower extremity weakness, resulting in impaired ambulation. Neurologic examination demonstrated an upper motor neuron-predominant pattern involving the hip flexors, knee extensors, and foot dorsiflexors. KPS was rated as 80. Brain MRI revealed a left frontoparietal rim-enhancing lesion measuring 3 × 4 × 2.9 cm (Figure 1F-F′). She underwent subtotal resection due to lesion location, with placement of carmustine (Gliadel) wafers. Histopathologic examination showed a pleomorphic epithelioid neoplasm with a high Ki-67 proliferative index (20%) and immunoreactivity for pan-NTRK and GFAP (Figure 1G-J). Targeted sequencing identified a *TERT* promoter mutation (c.-146C > T), no EGFR amplification, and a *KANK1::NTRK2* fusion also involving exon 14 of *NTRK2*, preserving the full NTRK2 kinase domain (Figure 2B). The *MGMT* promoter was methylated. The patient received 60 Gy radiation therapy in 30 fractions with concurrent temozolomide.

Approximately 2 months post-diagnosis, she developed worsening musculoskeletal pain and plateaued neurologic improvement. After goals-of-care discussion and consideration of prognosis, she discontinued disease-directed therapy after 2 cycles of temozolomide and transitioned to hospice care out of state. She died 6 months after diagnosis.

### Aggressiveness of *KANK1::NTRK2* GBMs

These cases underscore the highly aggressive clinical course of *KANK1::NTRK2* fusion GBMs, marked by rapid progression, early recurrence, and poor survival despite standard therapy. In case #1, radiographic recurrence occurred within 1 month of initial resection (Figure 3C), despite prompt post-operative radiation and temozolomide therapy, necessitating repeat surgery on post-operative day 32. Histopathologic examination of the second excision specimen again showed a GBM (Figure 3C′). Imaging following the second procedure revealed the extensive infiltrative disease involving the brainstem (Figure 3D), which was confirmed at autopsy (Figure 3H). After the second resection, the patient survived slightly more than 6 months with progression of disease on surveillance imaging (Figure 3E and F), with increasing cranial nerve dysfunction and lethargy, despite treatment with the NTRK inhibitor entrectinib. At autopsy, while the original temporal lobe tumor site showed mostly reactive changes (Figure 3G-G′), diffuse infiltrative glioma tumor cells were identified in the brainstem and in the leptomeningeal space (Figure 3H-H′).

Similarly, case #2 exhibited rapid clinical decline, with progression of neurologic symptoms and limited functional recovery despite surgical resection and initiation of chemoradiation. The patient transitioned to hospice care within 2 months of diagnosis and died approximately 6 months after diagnosis. Collectively, these findings suggest that *KANK1::NTRK2* fusion GBMs represent a highly aggressive molecular subtype characterized by early progression, diffuse infiltrative spread, and limited response to conventional therapies.

### Spatial Proteogenomic Profiling of *KANK1::NTRK2* GBMs

**Figure 4.**
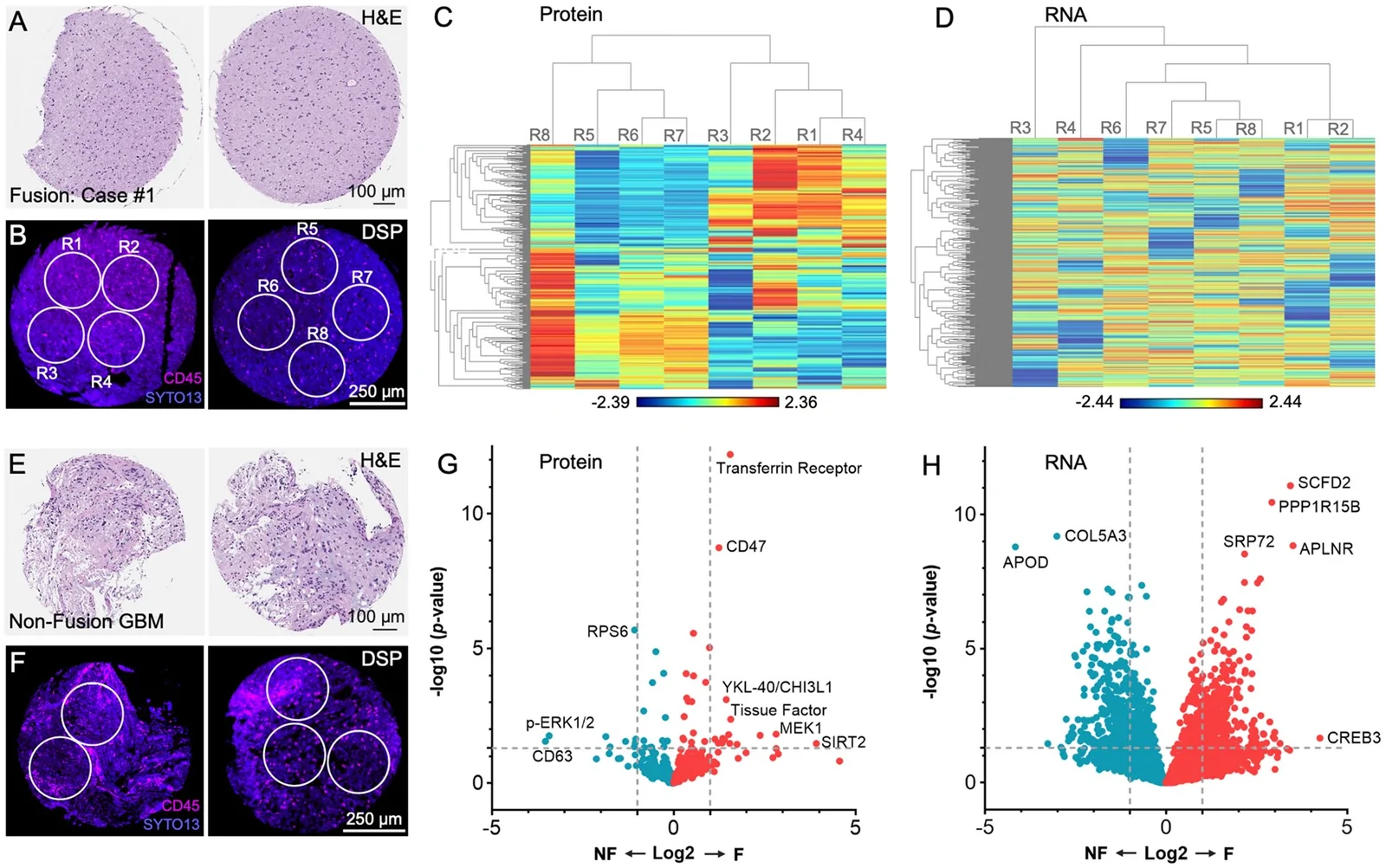
Spatial proteogenomic profiling of *KANK1::NTRK2* fusion GBM. Tissue cores from case #1 are shown with (A) H&E staining and (B) immunofluorescence DSP labeled with CD45 (magenta) and SYTO13 (blue), highlighting the 8 ROIs (R1-R8) selected for spatial proteogenomic analysis. (C, D) Heatmaps showing unsupervised hierarchical clustering of (C) protein and (D) transcript expression across the 8 ROIs from case #1. (E) H&E staining and (F) corresponding DSP image of a non-fusion GBM control case. (G, H) Volcano plots showing differential expression of (G) proteins and (H) RNA between the non-fusion GBM and the *KANK1::NTRK2* fusion tumor (case #1). The *y*-axis represents the − log_10_  *P*-value, and the *x*-axis represents the log_2_. Horizontal dashed line indicates statistical significance at *P* < 0.05, and vertical dashed lines indicate a 2-fold change threshold. Targets downregulated and upregulated in the *KANK1::NTRK2* fusion tumor are shown in blue and red, respectively.

To further characterize KANK1::NTRK2 GBMs, we performed GeoMx DSP on the initial resection specimen from case #1 using the Whole-Transcriptome Atlas (WTA; >18,000 transcripts) and the Immuno-Oncology Proteome Atlas (IPA; > 570 proteins), enabling matched RNA and protein profiling within the same ROI. ROIs were defined as intermediate regions containing both neoplastic cells and the surrounding microenvironment (Figure 4A and B).

Unsupervised hierarchical clustering demonstrated stronger ROI grouping by anatomical core in the proteomic dataset compared with the transcriptomic dataset, indicating greater spatial coherence at the protein level (Figure 4C and D). Proteomic profiling revealed increased expression of key regulators of tumor proliferation and invasion, including hexokinase 1, S100B, CD90, MEK1/2, ERK1/2, and VEGFR. These findings support activation of canonical NTRK2 downstream signaling pathways, particularly MAPK/ERK, along with PI3K-associated metabolic reprogramming coupled with VEGF-mediated angiogenesis (Supplementary Figure S1A). Consistent with genomic analysis demonstrating the preservation of the NTRK2 kinase domain, IHC validation confirmed increased expression of S100B, phospho-MEK1, and phospho-ERK1/2 in both fusion cases. Together, these results support sustained MAPK pathway activation downstream of the *KANK1::NTRK2* fusion (Supplementary Figure S2).

Spatial transcriptomic profiling demonstrated enrichment of genes involved in cellular metabolism, transcriptional and translational regulation, and microenvironment markers, including *MZT2B*, *GFAP*, and *MBP*, consistent with a highly proliferative and metabolically active tumor state. Notably, upregulation of *ADAM15* (A disintegrin-like and metalloproteinase 15), a metalloproteinase involved in ECM remodeling, suggests that NTRK2-driven signaling may extend beyond proliferative pathways to actively reshape the tumor microenvironment (Supplementary Figure S1B).

To contextualize these findings, we performed comparative analysis with a non-fusion GBM processed on the same platform at the same time. Despite similar clinical features—male sex, and tumor-associated seizures—the *KANK1::NTRK2* fusion case (temporal lobe; ∼7 months survival) exhibited a markedly more aggressive phenotype compared to the non-fusion case (frontoparietal; ∼29 months survival). Both tumors shared a similar genomic background of IDH WT status and were MGMT unmethylated. Unsupervised clustering of both proteomic and transcriptomic datasets segregated ROIs by case, indicating distinct oncogenic programs (Supplementary Figure S3A and B). Consistent with this separation, the fusion GBM demonstrated enrichment of proteins associated with tumor growth, invasion, angiogenesis, and therapeutic resistance, including transferrin receptor, CD47, tissue factor, CD90, CHI3L1, MEK1, adenosine receptor A2A, PAK1, SIRT2, MX1, c-MET, CCR6, CD15, MMP3, and active YAP1. In contrast, the non-fusion GBM exhibited relatively higher expression of proteins associated with alternative metabolic and stress-response pathways, such as RPS6, CD63, phospho-ERK1/2, HSP27, SIRT1, GLB1, vimentin, sonic hedgehog, and CPT1A (Figure 4G).

**Figure 5.**
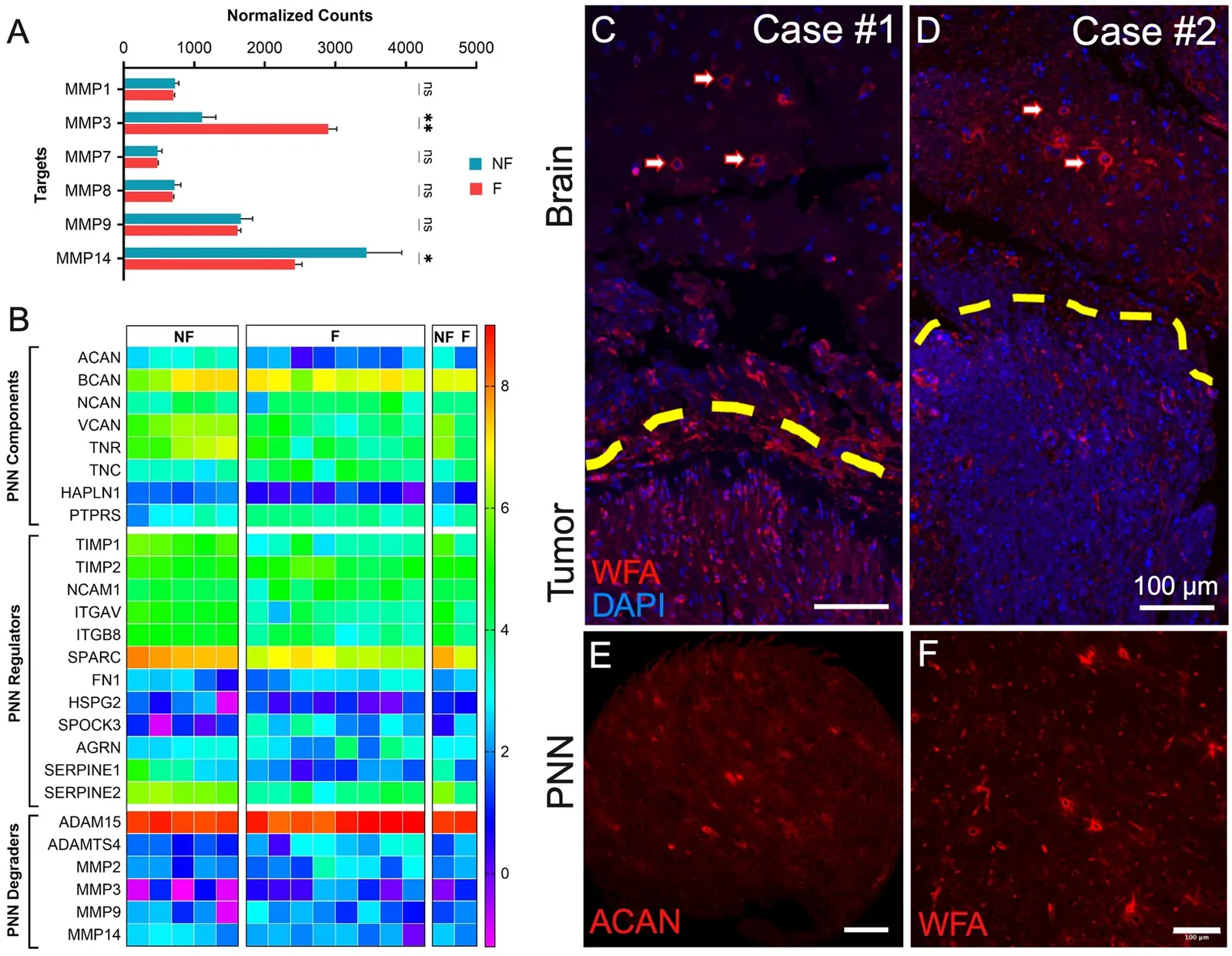
PNN organization in *KANK1::NTRK2* fusion GBM. (A) Bar plots showing the normalized MMP protein counts in non-fusion GBM (NF; blue) and *KANK1::NTRK2* fusion GBM (F; red). Error bars indicate the standard error of the mean. Statistical significance is indicated as *P *< 0.05 (*), *P *< 0.001 (**), and ns for non-significance, determined by Mann-Whitney test with Holm-Šidák’s multiple comparison test. (B) Heatmap showing the expression of PNN-related transcripts in non-fusion GBM (NF) compared to *KANK1::NTRK2* fusion GBM (F). Red indicates increased expression, whereas magenta indicates decreased expression in the profiled GBM cases. (C, D) Representative confocal images of (C) case #1 and (D) case #2 immunolabeled with *Wisteria floribunda* agglutinin (WFA; red) and DAPI (blue), highlighting tumor and adjacent brain regions. The tumor boundary is marked by a yellow dotted line, and arrowheads indicate the neurons with intact PNN. Scale bar: 100 µm. (E, F) High-resolution images of (E) case #1 tissue microarray core stained with aggrecan antibody and (F) case #1 tumor leading edge region demonstrating *Wisteria floribunda* agglutinin immunofluorescence in the adjacent brain tissue. Both stains demonstrate similar morphology of reticulated peri-soma structures consistent with perineuronal nets. Scale bars 100 µm.

At the transcriptomic level, the *KANK1::NTRK2* fusion GBM showed enrichment of pathways related to cell cycle progression, chromatin organization, and DNA replication, alongside downregulation of ECM-related pathways, including extracellular proteoglycans and chondroitin sulfate biosynthesis and metabolism (Figure 4H and Supplementary Figure S3C). Together with proteomic findings of increased ECM remodeling enzymes, including MMP3 and MMP14, these hypothesis-generating data from a single case suggest that constitutive NTRK2 signaling may promote coordinated ECM degradation and remodeling (Figure 5A, Supplementary Figures 3C and 4).

### PNN Degradation in *KANK1::NTRK2* GBMs

This ECM remodeling signature promoted further investigation of PNNs, a specialized ECM within the brain tumor microenvironment that encases GABAergic fast-spiking interneurons and has been associated with brain tumor-related epilepsy^20^ as well as inhibition of glioma invasion and proliferation and cortical network activity in mouse glioma xenograft models.^21^^,^^22^ Comparative spatial transcriptomic analysis of PNN-associated pathways demonstrated that the *KANK1::NTRK2* fusion GBM displayed a shift toward ECM degradation (Figure 5B, Supplementary Figure S3C), suggesting disruption of PNN integrity as a potential mechanism facilitating tumor invasion and increased proliferation via growth signaling through neuronal excitability.^23^ In contrast, the non-fusion GBM exhibited relative upregulation of PNN structural components (eg, *ACAN*, *HAPLN1*, *TNR*) and associated regulatory molecules (eg, *SPARC*, *SERPINE1*), alongside downregulation of PNN-degrading enzymes, including matrix metalloproteinases (eg, *MMP3*, *ADAMTS4*).

Additionally, to broaden our analysis beyond our single comparator GBM, we surveyed publicly available GBM databases, including IvyGAP, TCGA, GBMap, GBM-space, and the Brain Protein Atlas. We identified 3 neoplasms in the IvyGAP bulk RNAseq dataset that were matched for *MGMT*-unmethylated status and had no EGFR amplification where transcriptomic profiling had been undertaken in the “leading edge” region of the tumor, matching our GBM case #1 dataset. We compared the same suite of PNN-associated transcripts as in our non-fusion GBM case, and found a similar pattern of increased expression of PNN components and decreased expression of PNN degraders compared to the fusion GBM (Supplementary Figure S5).

To validate these findings at the tissue level, we performed immunolabelling within the tumor microenvironment. *Wisteria floribunda* agglutinin (WFA) staining demonstrated marked depletion of PNNs in brain regions immediately adjacent to tumors in both *KANK1::NTRK2* fusion GBM cases (Figure 5C and D), with relative preservation at distances approximately 200 µm from the tumor margin (Figure 5E and F) which was similar in morphology and density to the Aggrecan (*ACAN*) staining performed on the tissue core from GBM case #1. Together, these observations link *NTRK2*-driven signaling, ECM remodeling, and localized PNN degradation to the invasive phenotype of *KANK1::NTRK2* tumors.

## Discussion

In recent years, advanced molecular profiling platforms have substantially advanced our understanding of the molecular heterogeneity of GBMs, providing deeper insights into the biological basis of clinical variability. Comprehensive molecular characterization has demonstrated that specific genetic alterations define biologically and clinically distinct subsets of high-grade gliomas. A notable example is the identification of *IDH* mutation status, which is strongly associated with prolonged survival and has fundamentally reshaped the classification and clinical management of diffuse gliomas.^24^ Conversely, the study of genomic alterations associated with particularly poor outcomes may elucidate mechanisms underlying rapid recurrence, local invasion, and aggressive clinical behavior, while also revealing potential therapeutic targets.

In this study, we identified *KANK1::NTRK2* fusions in 2 patients with GBM who experienced highly aggressive clinical courses. Although rare, this fusion has been described in a limited number of high-grade gliomas and appears to be associated with extensive tumor infiltrative behavior and reduced survival. To date, 7 cases of high-grade gliomas harboring *KANK1::NTRK2* have been described in the literature, spanning a range of histologic subtypes (Table 1), including 4 pediatric patients, 2 young adults, and 1 older adult. Notably, both cases in our cohort also harbored *TERT* promoter mutations. Prior studies of *NTRK* fusion-positive tumors have shown that co-occurring canonical oncogenic drivers are uncommon and that overall mutational burden is typically low,^9^ supporting the hypothesis that *NTRK* fusions frequently act as primary oncogenic drivers in these tumors.

Recognition of *KANK1::NTRK2* fusions as a potential driver alteration has important therapeutic implications. Targeted inhibition of NTRK signaling has demonstrated promising clinical efficacy across multiple tumor types. In a cohort of 119 patients with primary brain tumors or brain metastases harboring *NTRK* fusions, treatment with larotrectinib resulted in durable responses.^25^ Similarly, entrectinib has shown activity in CNS metastases arising from *NTRK* fusion-positive malignancies.^26^^,^^27^ These findings highlight the importance of identifying *NTRK* fusions in gliomas, particularly tumors exhibiting unusually aggressive clinical behavior. Only one of our patients (case #1) was initiated on entrectinib therapy, but he did demonstrate clinical stability and no radiographic progression for a period of 6 months while on the medication. Unfortunately, he began to experience rapid functional decline from tumor growth in the brainstem which did not respond to an increased dose of entrectinib, and he elected to stop treatment. It is unclear why his response was less robust compared to responses of patients reported in Table 1.

To further investigate the biological consequences of the *KANK1::NTRK2* fusion, we performed spatial proteogenomic profiling to characterize downstream transcriptional programs and signaling pathway activation. ROI clustering occurred more robustly with the proteomic data than the transcriptomic data. In a previous study using this proteogenomic atlas in glioblastoma, there was discordance in mRNA and protein expression levels.^18^ There are several possible reasons, including greater protein stability. Indeed, Bonnett et al^18^ found in several instances that specific proteins, but not their corresponding mRNAs, were present in glioblastoma. Another group found that there was protein/RNA discordance for the classical subtype of GBMs but not the neural subtype.^28^

Relative to a fusion-negative GBM from a patient with a survival of 29 months, *KANK1::NTRK2* fusion-positive tumor regions showed upregulation of MAPK signaling and downregulation of ECM-associated components at both the mRNA- and protein-levels. These findings were corroborated by immunolabeling (Figure 5, Supplementary Figure S2). Such pathway enrichments are consistent with the canonical NTRK-driven signaling and are indicative of a highly proliferative, metabolically active, and invasive tumor phenotype.

Spatial transcriptomic analysis further identified *ADAM15* as one of the most highly expressed transcripts in both non-fusion and fusion GBMs. *ADAM15* has been implicated in promoting glioblastoma proliferation, angiogenesis, and metastasis through activation of protease-activated receptor 1 (*PAR1*).^29^ In addition to ADAM family members, other metalloproteases, including MMPs and ADAMTS, also play critical roles in ECM remodeling—a process central to glioma invasiveness. These proteases were likewise upregulated in *KANK1::NTRK2* fusion GBM, potentially contributing to its aggressive tumor phenotypes. Consistent with the well-described association between ECM remodeling and glioma invasiveness,^15^ we observed loss of PNNs in regions adjacent to the tumor infiltration. Together, these findings suggest that enhanced ECM degradation may facilitate rapid progression, local invasion, and tumor cell migration, highlighting ECM remodeling as a potential marker of invasiveness. We then surveyed publicly available GBM databases and identified 3 cases where the leading edge region of the tumor was profiled to build a larger comparator group. While this analysis had significant limitations, it supported the finding that relative to presumed non-fusion GBMs, PNN-related genes in the fusion GBM were downregulated whereas PNN-degrader genes in the fusion GBM were upregulated (Supplementary Figure S5).

Our spatial proteogenomic analysis of a *KANK1::NTRK2* fusion GBM provides important insights into the potential molecular drivers that may contribute to tumor proliferation and invasion in these rare tumors. However, several limitations should be acknowledged. First, this study describes a single case, limiting generalizability of our findings. Given the rarity of *KANK1::NTRK2* fusion GBMs, validation in additional cases will be essential, perhaps through multi-center collaboration. Second, we did not perform functional experiments to directly evaluate the relationship between the *KANK1::NTRK2* fusion protein and ECM remodeling. Consequently, our findings should be considered hypothesis-generating rather than evidence of causality. Third, our comparative analyses were constrained by the high cost of spatial transcriptomic and proteomic profiling and by our focus on the infiltrative tumor edge. Ideally, comparator cases would also be matched for molecular subtype, *MGMT* methylation status, age, gender, brain region, and clinical outcome. While we attempted to generate a larger comparator group using publicly available leading-edge transcriptomic data, this comparison was highly limited due to potential differences in tumor classification and unclear *KANK1::NTRK2* status. Future studies with larger cohorts, matched comparator specimens, and quantitative analyses of peritumoral ECM and functional assays—including in vitro models for PNN degradation and pharmacologic or genetic inhibition of *NTRK* signaling—will be needed to determine whether the observed associations reflect a causal role for the *KANK1::NTRK2* fusion in aggressiveness of GBMs.

In summary, *KANK1::NTRK2* fusions represent a rare but recurrent molecular alteration in high-grade gliomas that may be associated with aggressive clinical behavior. In this study, we performed spatial transcriptomic and proteomic analysis of a GBM harboring a *KANK1::NTRK2* fusion to reveal molecular-level changes that drive malignant behavior, including enrichment of transcriptional programs linked to invasion and tumor progression and degradation of key components of PNNs in proximity to the tumor. Detection of this fusion carries important prognostic and therapeutic implications, particularly given the availability of *NTRK*-targeted therapies such as larotrectinib and entrectinib. Continued investigation of *NTRK*-targeted therapies in primary brain tumors, alongside improved molecular characterization of rare fusion-driven gliomas, may eventually expand treatment options for patients with these highly aggressive malignancies.

## Supplementary Material

vdag219_Supplementary_Data

## Data Availability

Data and images are available upon request to the corresponding author.
