# Supplementary material for: Perineuronal net degradation in aggressive glioblastomas with KANK1::NTRK2 fusions
Source: Neurooncol Adv. 2026 Aug 20;8(1):vdag219. doi: 10.1093/noajnl/vdag219 (PMC13552376; doi:10.1093/noajnl/vdag219)
Supplement: Supplementary file 1 [file vdag219_supplementary_data.zip › KANK1_NTRK2 Supplementary figures REVISED.docx]

**Perineuronal net degradation in aggressive glioblastomas with *KANK1::NTRK2* fusions**

**Supplemental data:**

**Figure 1. Enriched molecular targets in *KANK1::NTRK2* fusion GBM.** Bar plots showing the top 50 enriched (**A**) proteins and (**B**) RNA across eight ROIs from case #1 based on spatial proteogenomic profiling. The y-axis represents mean normalized counts, while targets are shown on the x-axis. Protein expression data were normalized to house-keeping controls, whereas RNA expression data were normalized using the Q3 method. Error bars indicate the standard error of the mean.

**Figure 2.** **IHC analysis of** ***KANK1::NTRK2* fusion GBMs.** Representative IHC staining from (**A-C**) case #1 and (**D-F**) case #2 demonstrating immunoreactivity for (**A**, **D**) S100B, (**B, E**) phospho-MEK1, and (**C**, **F**) phospho-ERK1/2.

**Figure 3. Spatial proteogenomic profiling of GBMs.** (**A-B**) Unsupervised hierarchical clustering heatmaps of (**A**) protein and (**B**) transcript expression across five ROIs from non-fusion GBM (NF; orange) and eight ROIs from *KANK1::NTRK2* fusion GBM case #1 (F; blue). (**C**) Heatmap showing the expression of PNN-related transcripts in non-fusion GBM (NF) compared to *KANK1::NTRK2* fusion GBM (F). Red indicates increased expression, whereas blue (**A** and **B**)/magenta (**C**) indicates decreased expression in the profiled GBM cases.

**Figure 4. Spatial proteogenomic profiling of GBMs.** Pathway enrichment analysis displaying normalized enrichment scores for non-fusion (NF) compared with the profiled *KANK1::NTRK2* fusion GBM (F). Red indicates increased pathway activity, whereas blue indicates decreased activity in the profiled *KANK1::NTRK2* fusion GB.

***Supplemental Figure 5. PNN transcriptomic analysis of publicly available matched GBM data.*** *(****A****) We identified total of nine neoplasms in the IvyGAP dataset where molecular profiling was matched for MGMT-unmethylated status and no EGFR amplification. Highlighted (green) three cases with transcriptome data from the “leading edge” were compared with the fusion GBM dataset. (****B****) Heatmap showing comparative gene expression of PNN-related transcripts between the KANK1::NRTK2 GBM (F) and three IvyGAP tumors revealing a relative increase in PNN components and a relative decrease in PNN degraders relative to the fusion GBM for the IvyGAP tumors, similar to the pattern observed in the non-fusion GBM in our dataset.*
